# Capillary Bedside Blood Glucose Measurement in Neonates: Missing a Diagnosis of Galactosemia

**DOI:** 10.4274/jcrpe.1805

**Published:** 2015-03-05

**Authors:** Mehmet Nuri Özbek, Murat Öcal, Sibel Tanrıverdi, Birsen Baysal, Ahmet Deniz, Kahraman Öncel, Hüseyin Demirbilek

**Affiliations:** 1 Diyarbakır Training and Research Hospital, Clinics of Pediatrics, Diyarbakır, Turkey; 2 Hacettepe University Faculty of Medicine, Department of Pediatric Endocrinology, Ankara, Turkey

**Keywords:** Pseudohyperglycemia, Galactosemia, conjugated bilirubinemia, capillary blood glucose

## Abstract

A number of factors may lead to inaccuracy in measurement of capillary blood glucose with a glucometer. Measurement of other carbohydrate molecules such as galactose and fructose along with glucose can potentially be a cause of error. We report a newborn patient who was referred to our hospital with conjugated bilirubinemia, hepatomegaly and high capillary blood glucose levels measured with a glucometer. Simultaneous biochemical measurements revealed normal blood glucose levels. Further investigation led to a diagnosis of classical galactosemia. Capillary blood glucose level measured with glucometer also dropped to normal values following cessation of breastfeeding and initiation of feeding with a lactose-free formula.

## INTRODUCTION

Frequent bedside capillary blood glucose measurements using a glucometer provide early detection and management of hypo- and hyperglycemia. Following the report of the “Diabetes Control and Complications Trial” which showed the role of intensive insulin regimen and frequent blood glucose measurement on improvement of glycemic control and thereby decreasing the complications of diabetes mellitus, self blood glucose monitoring using a glucometer has become a widely used procedure (1). Home blood glucose monitoring using glucometers increases awareness of hyper- and hypoglycemia in diabetic patients and promotes attainment of better hemoglobin A1c levels (2,3).

Hypo- and hyperglycemia are common metabolic complications also encountered in patients followed in neonatal and critical care units. Despite discrepancies between venous and capillary blood glucose measurements, availability of a simple, fast and practical method has made capillary blood glucose measurement a widespread blood glucose monitoring method (4,5,6). On the other hand, it is known that inaccuracies may be encountered in measurement with glucometer that may lead to diagnostic errors. Moreover, blood hemoglobin and lipid levels affect the capillary blood measurements. It is also known that the glucometer measures other carbohydrates such as galactose and fructose alongside with the blood glucose (7,8,9).

Herein, we report a case of pseudohyperglycemia in bedside capillary glucose measurement in a patient who had presented with conjugated bilirubinemia and was diagnosed as classical galactosemia; pseudohyperglycemia disappeared following weaning and initiation of feeding with a low-lactose formula.

## CASE REPORT

A 24-day-old male infant was referred to our endocrine and metabolic center because of conjugated bilirubinemia, elevated liver enzymes and hepatomegaly. The patient had been admitted to another hospital on the 7th day of life with complaints of poor feeding and jaundice.

The infant was born by spontaneous vaginal delivery at 40 weeks following an uneventful gestation. His birth weight was 3280 g. Parents were second cousins. The family history revealed a sibling who had the same clinical features and died on the 10th day of life from a condition of unknown etiology.

On physical examination at presentation, weight was 3660 g (10th-25th p), length was 54 cm (25th-50th p) and head circumference was 36 cm (5th-10th p). The sclera and skin were icteric. Lenticular opacity (cataract) and hepatomegaly were noted. Other system examinations were normal. Laboratory investigations performed at admission revealed elevated liver enzymes [alanine aminotransferase: 194 IU/L (N: 0-41); aspartate aminotransferase: 224 IU/L (N: 0-41)] and a high level of conjugated bilirubin (2.8 mg/dL). During follow-up, the bedside capillary blood glucose level was measured as 369 mg/dL, 367 mg/dL and 342 mg/dL on three occasions. Despite high capillary blood glucose, urinalysis showed no glucosuria. Biochemical glucose measurement was also normal (71 mg/dL). Test results for urinary reducing agents was (+++) and urinary sugar chromatography showed (++) galactosuria. Galactose level was 514.2 mg/dL (N<10.00) with an inappropriately low galactose-1-phosphate uridyl transferase level [0.86 U/g Hb (N>5.00)], as shown in Table 1.

**Figure 1:**
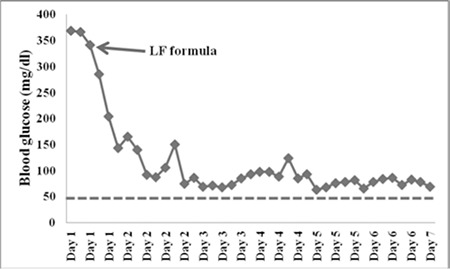
Capillary blood glucose measurements using a glucometer at admission and after starting on a lactose-free formula

Based on presence of conjugated hyperbilirubinemia, hepatomegaly, cataract, reducing agents in the urine, galactose on sugar chromatography and a low blood galactose-1-phosphate uridyl transferase activity, a diagnosis of classical galactosemia was considered. The infant was weaned from breastfeeding and started on a lactose-free formula. Following this change in diet, the bedside capillary blood glucose measurements decreased to the normal range and were even in the low normal range (Figure 1).

## DISCUSSION

Hyperglycemia in the neonatal period generally suggests neonatal diabetes mellitus (NDM). However, patients with NDM usually have intrauterine growth restriction and present with poor feeding, polyuria, signs and symptoms of dehydration and difficulty in weight gain. Laboratory findings confirm presence of hyperglycemia and glucosuria. Our patient, despite a high capillary blood glucose level, had no weight loss or glucosuria. Repeated measurements using glucometers of two different brands yielded high glucose levels (Figure 1). In addition to hyperbilirubinemia and hepatomegaly, urine tests revealed presence of urinary reducing agents. Cataracts were noted in the ophthalmological examination. These findings were all strongly suggestive of galactosemia. Therefore, breastfeeding was stopped immediately, while a detailed investigation was in progress. Following weaning from breastfeeding and starting the infant on a low-lactose formula, blood glucose level measured by bedside capillary measurement dropped to normal values.

There are various factors affecting the validity of bedside capillary blood glucose measurement using a glucometer. Inappropriate sampling, use of machine incompatible glucosticks or erroneous coding of glucosticks are common causes of error. Since glucosticks can measure other non-glucose carbohydrate molecules such as galactose and fructose, the presence of these substances may also change the test results. Elevated capillary blood glucose measurements have been reported in samples collected from the fingertip after peeling fruits (7).

In our case patient, biochemical measurement had not confirmed the high capillary blood glucose level, a finding which resulted from pseudohyperglycemia due to high galactose levels (Table 1). Indeed, in the user guide of both glucometers, it was indicated that a galactose level higher than 10 mg/dL may cause pseudohyperglycemia. We report the findings in this neonate to contribute to the literature, since reports of cases of pseudohyperglycemia due to galactosemia are rare (8,9).

Finally, although bedside capillary blood glucose measurement is a fast, simple and widely available method that provides early detection of hypo-/hyperglycemia, prompt diagnosis and immediate management of underlying pathologies, clinician should keep in mind the possibility and causes of pseudohypo/hyperglycemia. Once pseudohyperglycemia and conjugated bilirubinemia is detected in a neonate, the diagnosis of galactosemia should be suspected and confirmed after appropriate investigations.

## Figures and Tables

**Table 1:**
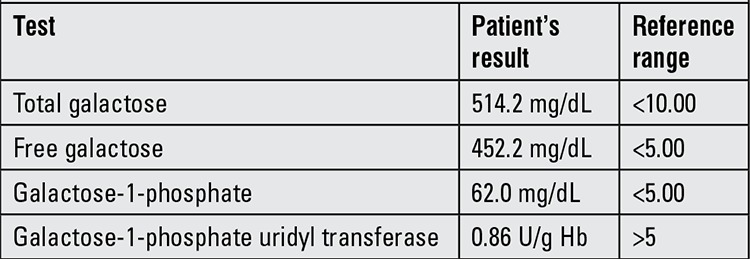
The results of the galactosemia panel of the patient
